# MicroRNA-145 Is Downregulated in Glial Tumors and Regulates Glioma Cell Migration by Targeting Connective Tissue Growth Factor

**DOI:** 10.1371/journal.pone.0054652

**Published:** 2013-02-04

**Authors:** Hae Kyung Lee, Ariel Bier, Simona Cazacu, Susan Finniss, Cunli Xiang, Hodaya Twito, Laila M. Poisson, Tom Mikkelsen, Shimon Slavin, Elad Jacoby, Michal Yalon, Amos Toren, Sandra A. Rempel, Chaya Brodie

**Affiliations:** 1 Davidson Laboratory of Cell Signaling and Tumorigenesis, Hermelin Brain Tumor Center, Department of Neurosurgery, Henry Ford Hospital, Detroit, Michigan, United States of America; 2 Everard and Mina Goodman Faculty of Life Sciences, Bar-Ilan University, Ramat-Gan, Israel; 3 Department of Public Health Sciences, Henry Ford Hospital, Detroit, Michigan, United States of America; 4 International Center for Cell Therapy and Cancer Immunotherapy (CTCI), Tel-Aviv, Israel; 5 Pediatric Hemato-Oncology, The Edmond and Lilly Safra Children’s Hospital, Sheba Medical Center, Tel-Hashomer and The Sackler School of Medicine, Tel-Aviv University, Tel-Aviv, Israel; 6 Barbara Jane Levy Laboratory of Molecular Neuro-Oncology, Department of Neurosurgery, Henry Ford Hospital, Detroit, Michigan, United States of America; University of Florida, United States of America

## Abstract

Glioblastomas (GBM), the most common and aggressive type of malignant glioma, are characterized by increased invasion into the surrounding brain tissues. Despite intensive therapeutic strategies, the median survival of GBM patients has remained dismal over the last decades. In this study we examined the expression of miR-145 in glial tumors and its function in glioma cells. Using TCGA analysis and real-time PCR we found that the expression of miR-145/143 cluster was downregulated in astrocytic tumors compared to normal brain specimens and in glioma cells and glioma stem cells (GSCs) compared to normal astrocytes and neural stem cells. Moreover, the low expression of both miR-145 and miR-143 in GBM was correlated with poor patient prognosis. Transfection of glioma cells with miR-145 mimic or transduction with a lentivirus vector expressing pre-miR 145 significantly decreased the migration and invasion of glioma cells. We identified connective tissue growth factor (CTGF) as a novel target of miR-145 in glioma cells; transfection of the cells with this miRNA decreased the expression of CTGF as determined by Western blot analysis and the expression of its 3′-UTR fused to luciferase. Overexpression of a CTGF plasmid lacking the 3′-UTR and administration of recombinant CTGF protein abrogated the inhibitory effect of miR-145 on glioma cell migration. Similarly, we found that silencing of CTGF decreased the migration of glioma cells. CTGF silencing also decreased the expression of SPARC, phospho-FAK and FAK and overexpression of SPARC abrogated the inhibitory effect of CTGF silencing on cell migration. These results demonstrate that miR-145 is downregulated in glial tumors and its low expression in GBM predicts poor patient prognosis. In addition miR-145 regulates glioma cell migration by targeting CTGF which downregulates SPARC expression. Therefore, miR-145 is an attractive therapeutic target for anti-invasive treatment of astrocytic tumors.

## Introduction

Glioblastomas (GBM), the most malignant of the primary brain tumor, are characterized by increased proliferation, robust angiogenesis and invasion into the surrounding normal brain tissue [1]. Current treatments include surgery, radiation therapy and chemotherapy. Unfortunately, the prognosis of these patients remains extremely poor [1]–[3]. Limitations to therapy include the infiltrative nature of the tumors which prevents complete resection and contributes to tumor recurrence and the high resistance to radio- and chemotherapy of residual tumor cells and glioma stem cells (GSC) [4], [5]. Since tumor infiltration is a major reason for treatment failure [6], [7], the development of novel therapeutic strategies aimed at limiting or eliminating the invasive phenotype of these tumors could have a profound effect on patient outcome.

MicroRNAs (miRNAs) are small non-coding RNAs that negatively regulate gene expression by interacting with the 3′-UTR of mRNA leading to gene silencing by either degradation or repression of mRNA translation [8], [9]. Because miRNAs cause gene silencing by partial sequence homology, a single miRNA can have hundreds of targets and therefore regulate diverse cellular functions [8]. Indeed, deregulation of miRNA expression has been associated with various disorders including cancer [10], [11], and specific miRNAs have been reported to play major roles in tumor initiation and progression and in tumor migration and invasion [12], [13]. In GBM, various miRNAs such as miR-21 [14], miR-221/222 [15], miR-125 [16] and miR-10b [17], have been associated with the initiation and progression of glioblastoma and with their invasive nature. In contrast, miR-34a [18] and miR-326 [19] have been implicated as tumor suppressor miRNAs in these tumors.

miR-145 has been shown to be downregulated in various types of cancers and is considered a putative tumor suppressor miRNA [20]. Indeed, low expression of miR-145 has been reported in gastric [21], lung [22], breast [23] and prostate [24] cancers. Moreover, miR-145 inhibits cell growth by targeting c-Myc and IRS-1 [25], suppresses the pluripotent potential of embryonic and cancer stem cells by targeting OCT4, SOX2 and NANOG [26], [27] and regulates cell migration, invasion and metastasis by targeting ADAM17 [28], mucin1 [29], FSCN1 [30].

In this study we examined the expression of miR-145 in glial tumors and its effects on glioma cell functions. We found that miR-145 was downregulated in astrocytic tumors and oligodendrogliomas as compared to normal brain and that overexpression of miR-145 in glioma cells and gliomas stem cells decreased cell migration. Moreover, we identified connective tissue growth factor (CTGF) as a novel target of miR-145 that mediated its effects on cell migration via downregulation of SPARC.

## Results

### miR-145 Expression is Downregulated in Glial Tumors

**Figure 1:**
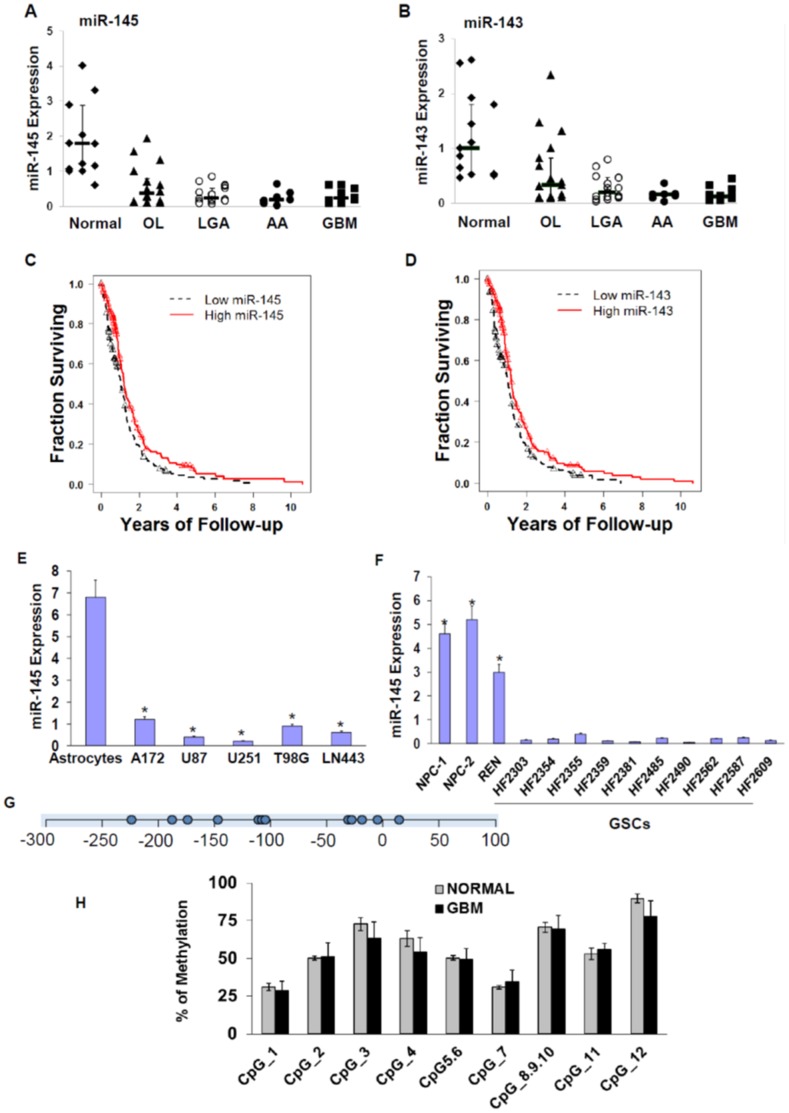
Expression of miR-145 in glial tumors, glioma cell lines and GSCs. The expression of miR-145 (A) and miR-143 (B) was determined in glial tumors using real-time PCR (p<0.001 for ANOVA F-tests and t-tests for all tumor samples as compared to normal brains). Data from individual human tissues are presented with the median and interquartile range noted. Results are normalized relative to the levels of S12 mRNA and are presented relative to a reference sample. (p<0.005 for all tumor specimens as compared to normal brains). Survival probabilities for patients with GBM expressing low and high levels of miR-145 (C) and miR-143 (D) were determined using Kaplan-Meier estimates and assessed with the log-rank statistic (P = 0.00359, P = 0.00194, respectively). The triangles indicate censored survival time points (i.e. patients alive at last follow-up). The expression of miR-145 in glioma cells as compared to human astrocytes (E) and in GSCs as compared to human neural progenitor cells (F) was determined using real time PCR. Methylation of miR-145 was determined in eight GBM specimens and eight normal brains. An amplicon starting 353 bp upstream pre-miR-145, and ending 78 bp downstream of pre-mir145 promoter, was used for the methylation analysis, covering 12 CpG sites. Zero is the position for the nucleotide starting pre-mir-145. Circles represent CpG site (G). Methylation was determined by Sequenom mass spectrometry detection for each CpG position in the specific amplicon (H). The results are representative of three independent experiments that gave similar results. *P<0.001.

miR-145 has been reported to be underexpressed in various types of tumors [21]–[24], however, its expression in astrocytic tumors has not been reported. We examined the expression of miR-145 in astrocytic tumors of different grades and in normal brain specimens using qRT-PCR. As presented in Figure 1A, the expression of miR-145 was significantly lower in all the glial tumors that were examined compared to normal brain. A similar expression pattern was found for miR-143 (Figure 1B) that is a cluster partner of miR-145 [31]. We found that among GBM specimens, elevated miR-145 (Figure 1C) and miR-143 (Figure 1D) expression was significantly associated with better patient survival (hazard ratio: 0.84, P = 0.0170; hazard ratio: 0.84, P = 0.0171, respectively). Dichotomizing expression demonstrated a 2.4 month increase in median survival times for tumors with high miR-145 or miR-143 levels (log-rank test P = 0.00359, P = 0.00194, respectively).

In addition, we found that the expression of miR-145 was lower in glioma cell lines as compared to normal human astrocytes (Figure 1E) and in GSCs as compared to human neural stem cells (NSCs) (Figure 1F).

miR-145 has been shown to be downregulated in prostate cancer by CpG hypermethylation in the promoter region [32]. To examine the role of this process in the downregulation of miR-145 in GBM, we studied the methylation status of the same region that was hypermethylated in prostate cancer and was associated with miR-145 downregulation (Figure 1G). We determined the methylation status of eight GBM specimens and eight normal brain samples and found no significant differences in the methylation status of the miR-145 promoter region between the GBM and control samples (Figure 1H).

### miR-145 Suppresses Glioma Cell Migration and Invasion

**Figure 2:**
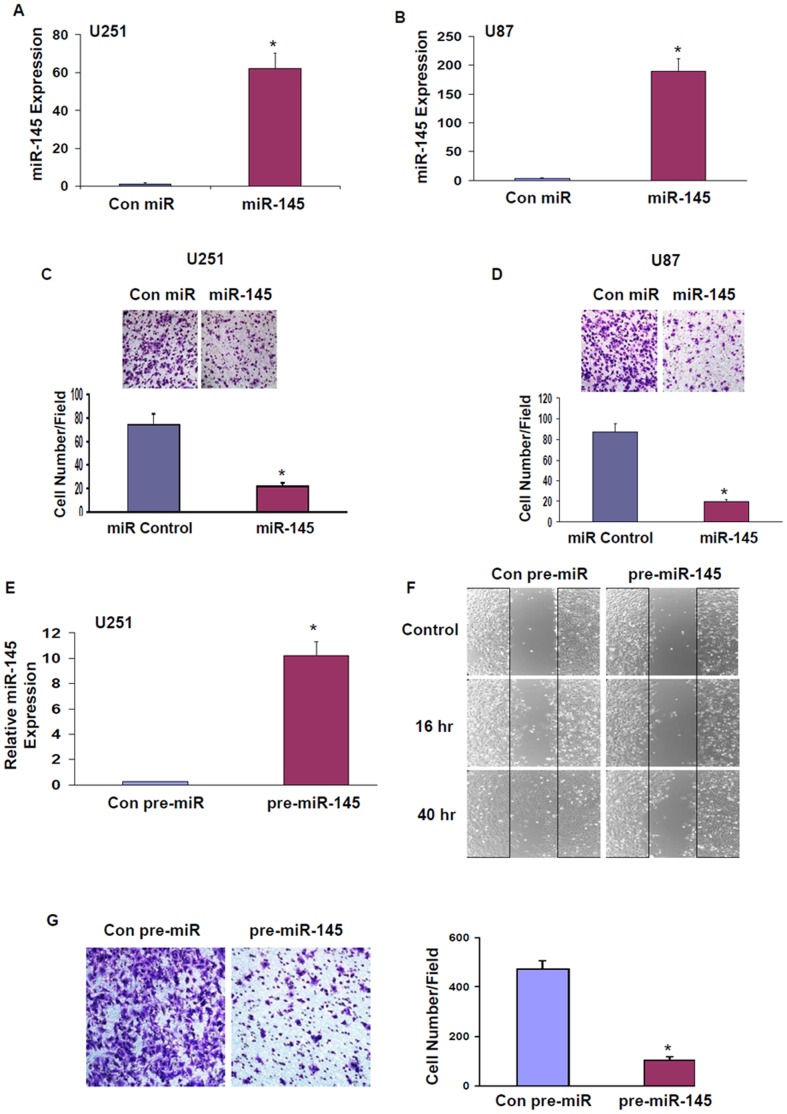
miR-145 decreases glioma cell migration. U251 (A) and U87 (B) cells were transfected with a miR-145 mimic and the expression level was determined using real-time PCR. Following 48 h, cell migration was determined for U251 (C) and U87 (D) cells using transwell migration assay. U251 cells were infected with a Tet-On lentivirus vector expressing pre-miR-145 or a control pre-miR. Stable clones of U251 cells were induced with doxycycline (0.5 µg/ml) and the expression of miR-145 was determined using real-time PCR (E). Cell migration in the induced cells was determined using wound healing migration assay (F) or transwell migration assay (G). The results are representative of three different experiments that gave similar results or are the mean ± SE of three different experiments. *P<0.001.

To further investigate the role of miR-145 in glioma cells we transfected the U251 (Figure 2A) and U87 glioma cells (Figure 2B) with a miR-145 mimic and examined the effect of the miR mimic on cell growth and migration. The expression of synthetic miR-145 did not exert a significant effect on cell proliferation (data not shown). In contrast, we found that the miR-145 mimic significantly decreased cell migration by about 60% in U251 cells (Figure 2C) and by about 75% in the U87 cells (Figure 2D) as determined by transwell migration assay.

We further examined the effect of miR-145 on glioma cell migration using an inducible lentivirus vector expressing pre-miR-145. Using qRT-PCR we demonstrated that infection of U251 cells with this plasmid and induction with doxycycline (0.5 µg/ml) increased the expression of the mature miR-145 in the cells (Figure 2E). Using wound healing assay (Figure 2F) and transwell migration assay (Figure 2G); we found that the induction of the pre-miR-145 significantly decreased U251 cell migration, whereas no significant effects were observed in cells expressing a control pre-miR.

**Figure 3:**
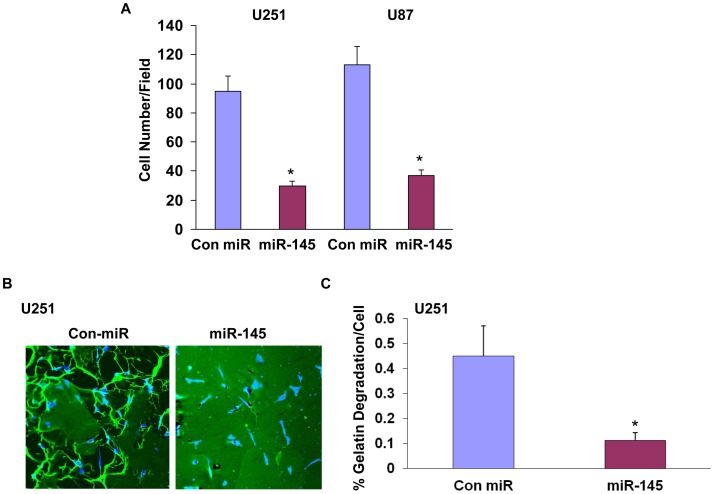
miR-145 mimic inhibits glioma cell invasion. miR-145 transfected U251 cells or U87 cells were plated on the top compartment of a Boyden chamber. After 8 h of incubation, cells that traversed the Matrigel-coated filters were stained and counted (A). U251 cells, transfected with miR-control or miR-145 mimic, were seeded on FITC-gelatin coated coverslips for 17 h. Cells were stained with an anti-cortactin antibody conjugated to Cy5 (B). The percentage of gelatin degradation per cell was determined in 10 fields (C). The results are the mean ± SE of three different experiments. *P<0.001.

In addition to its effect on cell migration, we also found that miR-145 decreased glioma cell invasion as indicated by matrigel invasion (Figure 3A) and gelatin degradation assays (Figure 3B, 3C). As presented in Figure 3A, transfection of the cells with miR-145 mimic significantly decreased the invasion of the U251 glioma cells. Moreover, using a gelatin degradation assay [33] we demonstrated that miR-145 inhibited matrix degradation by 4.5-fold after 2 days of transfection compared to cells transfected with a control miR (Figure 3B, 3C).

### miR-145 Targets CTGF

**Figure 4:**
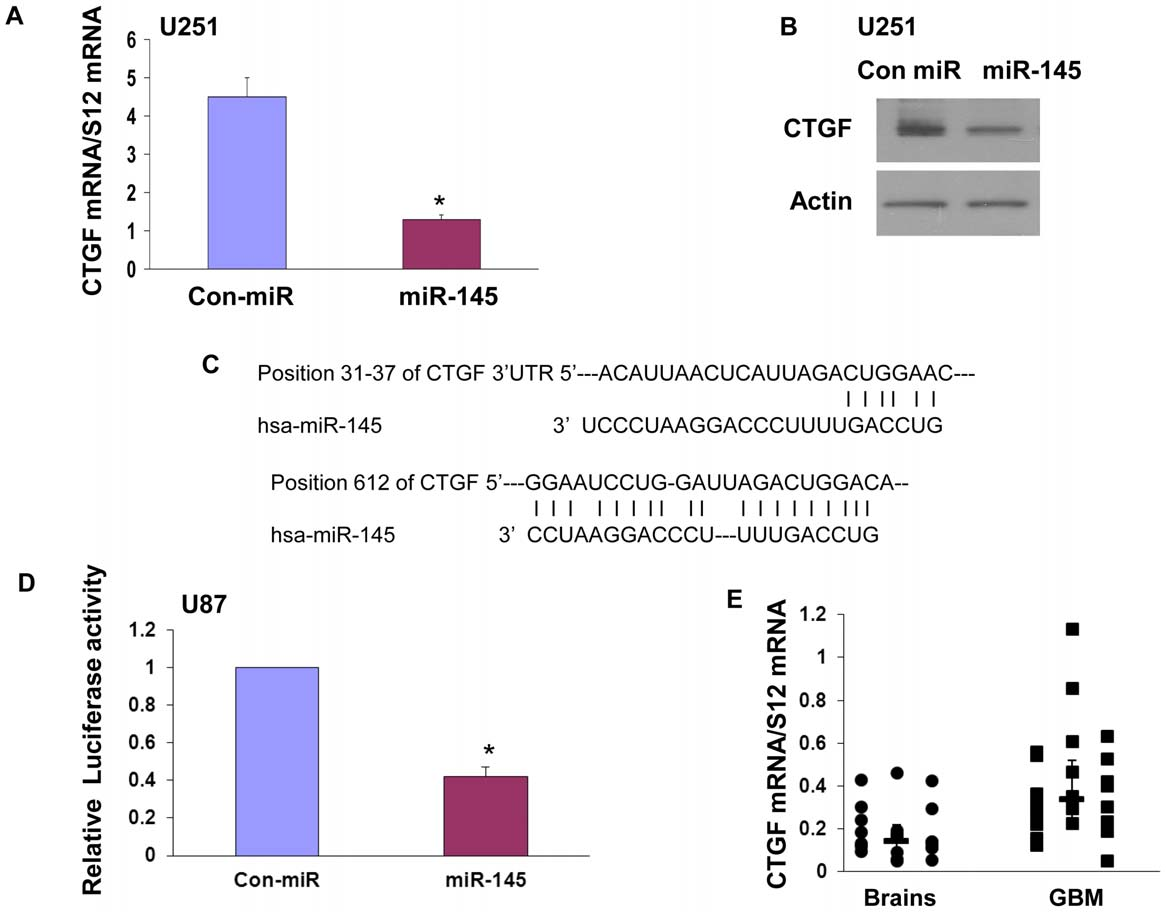
CTGF is a novel target of miR-145. U251 cells were transfected with miR-145 mimic for two days and the expression of CTGF was determined using real-time PCR (A) and Western blot analysis (B). Sequence alignment of CTGF 3′-UTR and miR-145 (C). Target sites of miR-145 in the CTGF 3′-UTR were analyzed using Targetscan and PicTar. (C). U87 cells were transfected with a CTGF 3′-UTR-luciferase plasmid followed by transfection with miR-145 mimic or control miR. The luciferase activity of the cells was determined after 48 h (D). The expression of CTGF was determined in normal brain and GBM specimens using real-time PCR. Data from individual human tissues are presented with the median and interquartile range noted. Data from individual human tissues are presented with the median and interquartile range noted. Age adjusted t-test, P = 0.001 (E). The results for panels A and D are the mean ± SE of four different experiments *P<0.001.

To delineate the mechanism by which miR-145 inhibited cell migration in glioma cells, we searched for miR-145 targets using the algorithms TargetScan4 and miRBase and found several putative miR-145 target genes that might play a role in cell migration. We focused on CTGF since it is a well known metastasis- and migration-promoting gene which is upregulated in several types of tumors, including gliomas [34]. Transfection of U251 cells with miR-145 mimic significantly decreased the expression of both CTGF mRNA (Figure 4A) by 72.2% and the CTGF protein by 79.8% (Figure 4B) in these cells compared to levels in control miR expressing cells. We then examined if CTGF is a direct target of miR-145. As presented in Figure 4C, the 3′-UTR domain of the CTGF mRNA is partially complementary to miR-145. Using a luciferase reporter system in which the 3′-UTR of the CTGF was cloned downstream of a luciferase gene (pEZX-MT01::CTGF-3′UTR) we demonstrated that miR-145 significantly decreased the luciferase activity of this construct in U251 cells, whereas a control miRNA had no significant effect (Figure 4D).

Finally, we examined the expression of CTGF in brain tumors and normal brain specimens. As shown in Figure 4E, CTGF expression was significantly elevated in GBM compared to the non-tumor samples. This finding is consistent with previous reports regarding CTGF expression in brain tumors [35], [36] and pancreatic cancer [37].

### The Targeting of CTGF by miR-145 Mediates the Inhibitory Effect of miR-145 on Glioma Cell Migration

**Figure 5:**
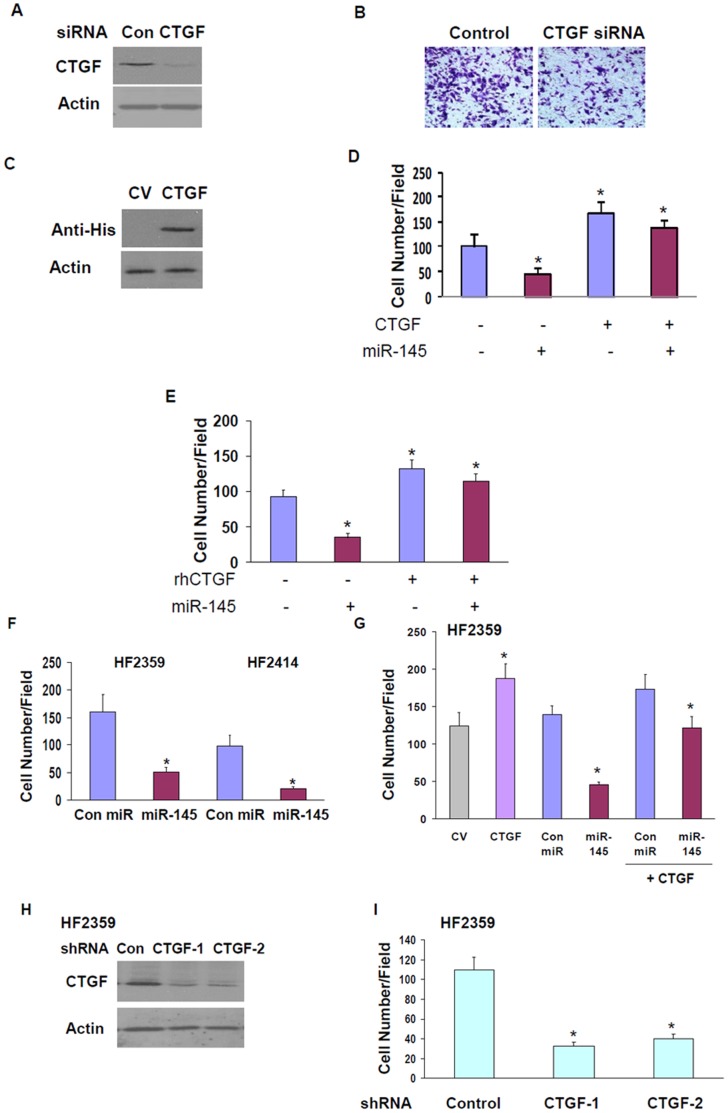
Targeting CTGF expression mediates the inhibitory effect of miR-145. The expression of CTGF was silenced in glioma cells using siRNA duplexes (A). Cell migration was determined after 48 h using transwell migration assay (B). CTGF plasmid lacking 3-‘UTR was overexpressed in U251 cells and exogenous CTGF expression was measured using Western blot analysis and anti-His antibody (C). CTGF plasmid lacking 3-‘UTR was overexpressed in U251 cells stably expressing pre-miR-145 or a control pre-miR and ell migration was determined using transwell migration assay (D). U251 cells expressing control pre-miR or pre-miR-145 were treated with human recombinant CTGF (100 nM) and cell migration was determined after 24 h using transwell migration assay (E). The HF2359 and HF2414 GSCs were dispersed to small spheroids and were transfected with either miR-145 mimic alone (F) or the HF2359 cells were transfected with miR-145 alone or together with CV or CTGF plasmids (G). Following 48 h, the spheroids were further dispersed into single cells and cell migration was determined using transwell migration as described in the methods (G). HF2359 cells were transduced with lentivirus vectors expressing two different CTGF shRNAs or control shRNA. The expression of CTGF was determined using Western blot analysis following 4 days of transduction (H) and cell migration was determined as described in the methods (I). The results are the means ± SE of three different experiments. *P<0.001.

We then examined the effect of CTGF on glioma cell migration and whether the decrease in CTGF expression mediated the inhibitory effect of miR-145 on cell migration. Small interfering RNA (siRNA) designed to target CTGF decreased the expression of CTGF protein by about 94% in the U251 cells (Figure 5A). In parallel, we found a significant decrease in cell migration 48 h following CTGF silencing (Figure 5B). Similar results were obtained using a lentivirus vector expressing a CTGF shRNA. Transduction of the U251 with the CTGF shRNA significantly decreased the migration of these cells as compared to cells transduced with a lentivirus vector expressing a control shRNA (data not shown).

To examine the role of CTGF in miR-145 effects, we employed a CTGF plasmid lacking the 3′-UTR. Overexpression of this plasmid increased CTGF protein expression (Figure 5C) and increased cell migration by about 70% (Figure 5D). Moreover, this overexpression abrogated the inhibitory effect of miR-145 on cell migration (Figure 5D). In addition, we examined the effect of a recombinant CTGF protein on cell migration and on miR-145 effects. Treatment of the U251 cells with 100 ng/ml of the recombinant CTGF protein increased cell migration by about 40% (Figure 5E). Transfection of the U251 cells with miR-145 in the presence of the CTGF protein abrogated the decreased cell migration induced by this miRNA (Figure 5E), further suggesting that the decreased cell migration induced by miR-145 is mediated by targeting of CTGF.

GSCs represent a subpopulation of GBM that recapitulates many molecular, biological and clinical aspects of their parental tumors [38]–[40]. In addition, these cells are highly infiltrative *in vitro* and *in vivo*. We therefore examined the effect of miR-145 in these cells and the role of CTGF in miR-145 effects. As presented in Figure 5F, transfection of the GSCs HF2359 and HF2414 with a miR-145 mimic significantly decreased the migration of the cells as determined by transwell migration assay. Similar to the results that were obtained with the U251 cells. transfection of the HF2359 GSCs with a CTGF plasmid lacking the 3′-UTR increased GSC migration and abolished the decrease in GSC migration induced by miR-145 (Figure 5G). In addition, transduction of the HF2359 GSCs with lentivirus vectors expressing two different CTGF shRNAs significantly decreased the expression of CTGF in these cells (Figure 5H) and the migration of these cells (Figure 5I) compared that of cells transduced with a control shRNA, further demonstrating the importance of CTGF in GSC migration.

### Silencing of CTGF Decreases the Expression of FAK and SPARC

**Figure 6:**
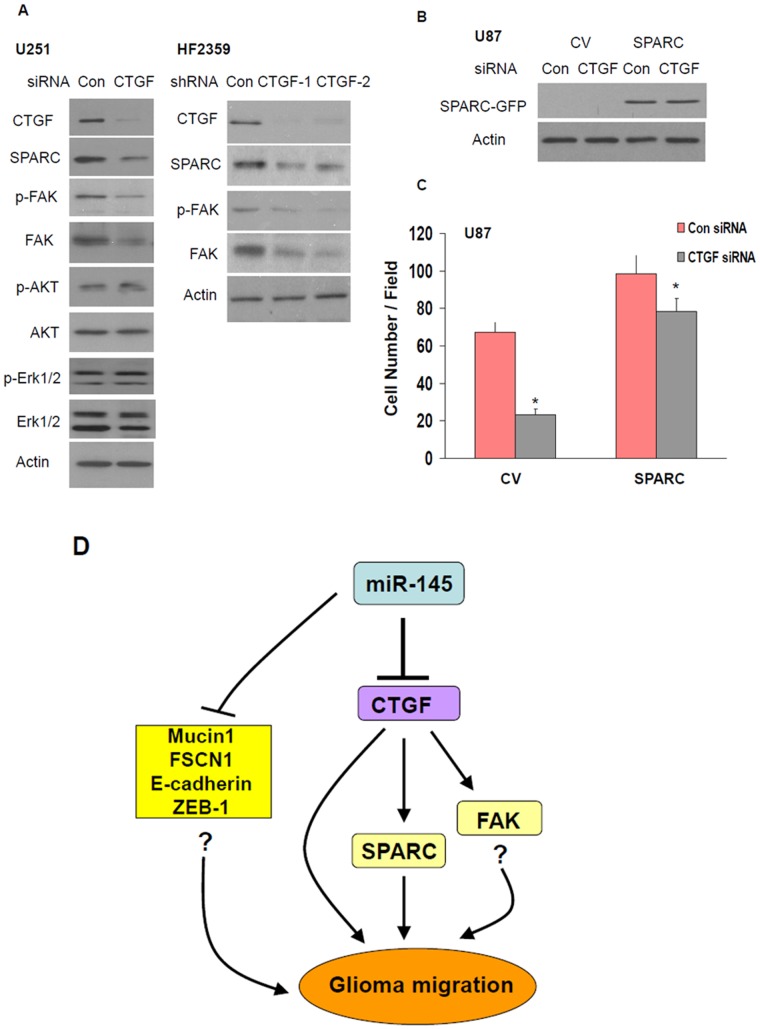
Silencing of CTGF decreases SPARC and FAK expressions. CTGF expression was silenced in U251 cells using siRNA duplexes. The expression of CTGF and SPARC and the expression and phosphorylation of FAK, AKT and ERK were determined in the control siRNA and CTGF siRNA transfected cells using Western blot analysis (A). HF2359 cells were transduced with lentivirus vectors expressing two different CTGF shRNAs or control shRNA. The expression of CTGF, SPARC, p-FAK, FAK and actin was determined using Western blot analysis following 4 days of transduction (A). Stable clones of U87 overexpressing SPARC-GFP or CV (GFP) were transfected with a control siRNA or CTGF siRNA duplex (B) and cell migration was determined using transwell migration assay (C). The results are representative of three different experiments which gave similar results (A, B) or are the means ± SE of three different experiments (C). P<0.001. A diagram of the effects of miR-145 on cell migration by targeting CTGF and additional pathways is presented (D).

To delineate the mechanisms by which CTGF decreases cell migration we examined the expression of several migration-related proteins in CTGF-silenced cells. We found that silencing of CTGF in the U251 cells decreased the expression and phosphorylation of FAK and the expression of SPARC (Figure 6A), two proteins that are strongly involved in the promotion of glioma cell migration [41], [42], but did not affect the phosphorylation and expression of AKT and Erk1/2. Similar results were obtained with U87 cells in which CTGF was silenced (data not shown) and in the HF2359 GSCs that were transduced with lentivirus vectors expressing two different CTGF shRNAs and a control shRNA (Figure 6A).

To further examine the role of SPARC in CTGF effects we employed a stable clone of SPARC overexpressing U87 cells [41] and a control (CV) clone (Figure 6B). As presented in Figure 6C, overexpression of SPARC increased cell migration as already described [43]. In addition, overexpression of SPARC abrogated the decreased cell migration induced by the silencing of CTGF (Figure 6C). Therefore, miR-145 can decrease cell migration directly by targeting CTGF and indirectly by decreasing the expression of SPARC downstream of CTGF. These results further emphasize the importance of miR-145 as a major regulator of glioma cell invasion by CTGF in addition to other published pathways in different cellular systems (Figure 6D).

## Discussion

GBMs are characterized by increased infiltration to adjacent brain tissue, which prevents complete tumor resection and contributes to tumor recurrence [4]–[7]. Therefore, understanding the mechanisms involved in glioma cell migration and identifying novel therapeutic targets are the focus of recent studies. In the present study we demonstrated that miR-145 is down-regulated in glial tumors and suppresses cell migration by inhibiting CTGF-mediated signaling pathways.

We found that the expression of miR-145 was down-regulated in astrocytic tumors and oligodendrogliomas compared to normal brain samples. Similarly, we found that glioma cell lines and GSCs expressed lower levels of miR-145 compared with normal human astrocytes and neural progenitor cells, respectively. Moreover, high expression of miR-145 predicted better patient prognosis. Our results are in accordance with recent studies indicating that miR-145 is down-regulated in various types of tumors including esophageal squamous cell carcinoma [44], colorectal cancer [45], breast [23], lung [22], liver and prostate cancer [32]. Moreover, the decreased expression of this miRNA is associated with poor prognosis in prostate cancer [24].

Recent studies have shown that the downregulation of several miRNA in some types of tumors is mediated by CpG island hypermethylation [46]. In prostate cancer, miR-145 downregulation is attributed to hypermethylation in its promoter region [32]. In contrast, we found that downregulation of miR-145 in the GBM specimens was not associated with hypermethylation of this region. Additional factors that can regulate miRNA expression include changes in signaling pathways and activation of specific transcription factors. Indeed, miR-145 expression has been reported to be regulated by TP53 [32], [47] which is frequently mutated in gliomas [48] and can therefore account for the decrease in miR-145 expression in these tumors. Recent studies demonstrated that C/EBP-β is a negative regulator of miR-145 via activation of the Akt pathway in breast cancer cells [49], and that EGFR downregulates miR-145 in colon cancer cells [50]. These pathways may potentially contribute to the downregulation of miR-145 in glial tumors. Additional studies are currently performed to delineate the mechanisms that are involved in the downregulation of miR-145 in gliomas.

Ectopic expression of miR-145 in glioma cells exerted only a minor negative effect on cell growth (data not shown), but significantly decreased glioma cell migration and invasion and GSC migration. Thus the decreased expression of miR-145 in these cells may be associated with the increased infiltration of astrocytic tumors. miR-145 has been shown to act as a tumor suppressor in various cellular systems by decreasing cell growth [25], apoptosis [51] and angiogenesis [52]. In addition, miR-145 has been reported to affect cell migration, invasion and metastasis in various types of tumors by targeting distinct cellular pathways [53], [54].

Several miR-145 targets have been recently identified in various cellular systems including IRS [25], E-cadherin [54], p70S6K1 [52], BNIP3 [55], YES [56], and STAT1 [56]. miR-145 has been reported to suppress the invasion and metastasis of breast cancer cells by directly targeting MUC1 [53] and that of esophageal cancer cells by deregulating FSCN1 [30]. In addition, miR-145 was reported to target the embryonic stem cell genes OCT4 and Nanog in embryonic stem cells and cancer stem cells [26]. We identified CTGF as a novel target of miR-145 in glioma cells and as a mediator of its effects on cell migration.

CTGF (CTGF/CCN2) is a member of the CCN family of proteins and is encoded by an early gene located on chromosome 6q23.1 [57]. CTGF is involved in extracellular matrix production, tumor cell proliferation, adhesion, migration, angiogenesis, and metastasis [58], [59]. CTGF has been recently reported to play a role in tumorigenesis and its expression is elevated in various tumors including acute lymphoblastic leukemias [60], esophageal squamous cell carcinomas [61], pancreatic cancers [61], [62] and prostate cancer [63]. CTGF expression is also increased in primary gliomas [64], and high levels of CTGF mRNA were directly correlated with advanced tumor stage [35], [64]. Furthermore, expression of CTGF in GBM is associated with tumor progression, drug resistance and glioma cell migration [36].

We found that miR-145 modulated CTGF expression by directly targeting the binding site within its 3′UTR. Furthermore, down-regulation of CTGF by siRNA inhibited glioma cell and GSC migration and conversely, overexpression of CTGF or treatment of glioma cells with a recombinant CTGF protein abrogated the inhibitory effects of miR-145 on glioma cell migration. Taken together, miR-145 inhibits glioma cell migration by directly targeting CTGF. However, since the inhibitory effect of miR-145 overexpression on cell migration is more robust than that of CTGF silencing, it is possible that miR-145 targets additional migration-related signaling pathways.

Silencing of CTGF in glioma cells and GSCs decreased the expression and phosphorylation of FAK and the expression of SPARC, two pathways that are implicated in cell migration [35], [37]. These results suggest that miR-145 targets CTGF, which in turn downregulates SPARC and FAK/pFAK, leading to suppression of cell migration. Activation of the FAK and Erk pathways by CTGF has been reported in chondrosarcoma cells [65]. Cross regulation of CTGF and SPARC was reported recently following fibrotic changes [66]. However, although both CTGF and SPARC play a major role in glioma cell migration [43], [65], [67], interaction of these proteins has not yet been reported.

Our finding that SPARC expression is regulated by CTGF and that overexpression of SPARC can abrogate the inhibitory effect of CTGF silencing on cell migration, demonstrates that these two proteins are associated in their effects on glioma cell migration. In a recent study, Edwards et al [67] reported that the pro-invasive effect of CTGF in GSCs is mediated by its interaction with integrin β1 and TrkA followed by the induction of the transcriptional repressor ZEB-1 and loss of E-cadhedrin. These studies further emphasize the importance of CTGF in glioma infiltration and the complex signaling pathways that mediate its effects (Figure 6D).

## Materials and Methods

### Materials

Primary antibodies were purchased from the following companies: anti-CTGF, anti-pFAK and anti-FAK from Abcam (Cambridge, MA); anti-SPARC from Santa Cruz Biotechnology (Santa Cruz, CA); and recombinant human CTGF protein from Abcam. PCR primers were purchased from Invitrogen. miR-145 mimic and CTGF siRNA were purchased from Dharmacon (Lafayette, CO). A lentiviral vector expressing miR-145 (inducible pMir-tet-145-GFP) was obtained from System Biosciences (SBI) (Mountain View, CA) and plasmids expressing CTGF (pReceiver-His) and luciferase-CTGF-3′UTR were obtained from GeneCopoeia (Rockville, MD).

### Human Tissue Specimens

Frozen human non-neoplastic brain tissue and human GBM specimens were obtained from the Department of Neurosurgery at Henry Ford Hospital. All human materials were used in accordance with the policies of the institutional review board at Henry Ford Hospital.

### TCGA Data Analysis

Processed miRNA expression data from the Agilent human miRNA 8x15K platform were obtained from the TCGA (Level 3, September 3, 2011) for 491 samples. For consistency, the origin of tissue was the brain, no prior tumor was recorded and the histopathology was noted to be untreated primary (de novo) GBM. There were 430 samples that met these criteria. Analysis of overall survival included Kaplan-Meier estimation of the effect of dichotomized miR expression (split at the median), compared with a log-rank test, and Cox proportional hazards models using miR expression as a continuous predictor.

### Bisulfite Treatment and Methylation Analysis

DNA was extracted according to the DNeasy kit, spin-column standard protocol (Qiagen, Maryland USA). Methylation analysis was performed on DNA produced from paraffin blocks of eight GBM specimens and eight normal brains. Bisulfite treatment was performed on 1 µg DNA using EZ DNA methylation Gold Kit (Zymo Research, Orange, CA), according to the Sequenom® protocol (Sequenom, San Diego, CA). Final elution of C-T converted DNA was with 50 µl H_2_O. CpG sites within mir-145 promoter sequence were searched using the USCS genome browser (http://genome.ucsc.edu). Amplicons and primers within the promoter area were designed using SequenomEpiDesigner (www.epidesigner.com). An amplicon starting 353 bp upstream pre-mir145, and ending 78 bp downstream pre-mir145, was used for the methylation analysis, covering 12 CpG sites. PCR amplification of the amplicon was carried out with 50 ng of bisulfite-treated DNA in a total volume of 25 µl with GoTaq buffer, 200 µM dNTPs, 5 mM MgCl_2_, 10 pmol of reverse and forward primers, and 2 U of GoTaq polymerase. PCR conditions were 1 cycle for 10 min at 95°C followed by 35 cycles for 20 s at 94°C, 30 s at 60°C and 1 min at 72°C, then later for 3 min at 72°C. PCR product was treated with Shrimp alkaline phosphatase and subjected to *in vitro* transcription into RNA and T-specific cleavage by RNase A, all according to Mass CLEAVE protocol (Sequenom®, San Diego, CA). For Sequenom mass spectrometry detection, samples were purified with 6 mg Resin and robotically dispensed onto 384-well spectro CHIPS by Mass ARRAY Nano dispenser (Sequenom, San Diego, CA). Results were analyzed by EpiTyper software.

### Isolation and Identification of GSCs

All human materials were used in accordance with the policies of the institutional review board at Henry Ford Hospital. The generation of GSCs and the enrichment of CD133+ cells and their characterization have been recently described [68], [69]. Briefly, GBM specimens were dissociated in 0.05% Trypsin/EDTA for 4 h at room temperature followed by incubation in DMEM/F-12 medium containing 0.7 mg/ml ovomucoid. The tissue was then triturated mechanically with a fire-narrowed Pasteur *cells* pipette and filtered through a 40-mm mesh. Cells were density centrifuged in Lympholyte-M and then maintained in neurosphere medium supplemented with 20 ng/ml EFG and 20 ng/ml FGFb. The enrichment of CD133+ cells was performed according to the MACS CD133 kit manual (Miltenyi Biotech). Spheroids were maintained in neurosphere medium and examined for the expression of CD44, Bmi-1, CD133, Musashi-1, Sox2 and nestin, self-renewal, and expression of astrocytic, oligodendrocytic and neuronal markers upon plating on poly-D-ornithine in serum-containing medium and for their tumorigenic potential in nude rats [68].

### Transfection and Infection of Glioma Cells

U87 and U251 cells were obtained from ATCC. All cells were cultured in DMEM supplemented with 10% FBS (Hyclone), 2 mM L-glutamine, and 100 µg/ml streptomycin-penicillin (Invitrogen) at 37°C under 5% CO_2_.

Transfection of glioma cells and GSCs with the miR-145 mimic and with the CTGF siRNA duplexes (SMARTpool, Thermo Scientific, Lafayette, CO) was performed by siIMPORTER (Millipore, Billerica, MA) in 6-well plates following the manufacturer’s recommendations. For overexpression of CTGF, cells were transfected using Amaxa Nucleofector (Lonza, Walkerville, MD) following the manufacturer’s protocol. A Tet-On lentiviral vector (System Biosciences, Mountain View, CA) expressing pre-miR-145 and lentivirus vectors expressing CTGF and control shRNAs were packaged and used to infect glioma cells according to the manufacturer’s protocol.

Stable clones of U87 cells overexpressing SPARC-GFP and GFP (CV) have been described before [41].

### Luciferase Assay

Cells were first transfected with appropriate plasmids in 12-well plates, and then were harvested and lysed for luciferase assay 48 h after transfection. Luciferase assays were performed by using a luciferase assay kit (Promega, Madison, WI) according to the manufacturer’s protocol. Renilla luciferase was used for normalization.

### Transwell Migration and Wound Healing Migration Assays

Transwell chambers (BD Biosciences, San Jose, CA) were used to determine the effect of miR-145 or CTGF on glioma cell and glioma stem cell (GSC) migration according to the manufacturer’s protocol [70]. In brief, transfected cells were harvested, resuspended in serum-free medium, and then transferred to the transwell chambers (25,000 cells per well). The chambers were then incubated for 3 h in culture medium with 10% FBS in the bottom chambers before analysis. The cells on the upper surface were scraped, whereas the migrated cells on the lower surface were fixed and stained with 0.05% crystal violet for 5 min. Finally, stained cells were counted under a microscope and the cell number was calculated. Wound healing assay was done using culture-inserts purchased from ibidi, LLC (Verona, WI) according to the manufacturer’s protocol.

### In vitro Invasion Assay

U251 and U87 cells were transfected with miR-145 mimic or control miRNA, and after 24-h plated on top of Matrigel-coated invasion chambers (24-well insert, 8-µm pore size; BD Biosciences) in a serum-free DMEM as previously described [71]. As a chemoattractant, DMEM containing 10% fetal calf serum was added to the lower chamber. After 20 h, noninvading cells were removed from the inner part of the insert by using a cotton swab. Fixation and staining of invaded cells were performed using a Diff-Quick differential staining set (Dade Behring, Inc., Deerfield, IL).

### Extracellular Matrix Degradation Assay

Fluorescently labeled fibronectin/gelatin-coated coverslips were prepared as recently described by Berdeaux et al [72]. Briefly, coverslips were coated with Oregon green 488-conjugated fibronectin/gelatin mixture (Sigma Chemical Co., St. Louis, MO) +2% sucrose, cross-linked for 15 min in 0.5% glutaraldehyde in PBS, and incubated for 3 min with 5 mg/ml NaBH_4_ in PBS. After washing with DMEM at 37°C, cells were plated on coated coverslips in DMEM and incubated for 17 h. For immunofluorescence staining the cells were fixed in 4% paraformaldehyde for 20 min and permeabilized with wash solution (0.1% Triton X-100, 1% bovine serum albumin in PBS) for 20 min. Cells were incubated with rabbit anti-cortactin polyclonal antibody (1∶300) for 45 min, washed three times with PBS and incubated with Cy5 anti-rabbit antibody for 1 hour. Coverslips were mounted on slides using anti-fade solution. For quantifying matrix degradation, images of 10 fields/10 mm^2^ per slide were acquired using eight-bit 512×512 pixel confocal Zeiss LSM510 microscope and AIM software. The percentage of degraded matrix per slide was analyzed using ImageJ software.

### Western Blot Analysis

Western blot analysis was performed as described [68]. Equal loading was verified using an anti-β-actin antibody.

### Real-time Quantitative PCR Analysis

Total RNA was isolated using TRIzol reagent (Invitrogen, Grand Island, NY) per the manufacturer’s protocol and 1 µg of RNA was used to synthesize cDNA by SuperScriptase III (Invitrogen) with random primers. To detect CTGF mRNA, the SYBR green method was used with primers CTGF (forward GGGAAATGCTGCGAGGAGT; reverse AGGTCTTGGAACAGGCGCTC); average level of S12 RNA was used as an internal control. Expression of miR-145 in cell lines or patient specimens was detected by the TaqMan stem-loop RT-PCR method. The primers and probes of the miR-145 and RNU6B endogenous control for TaqMan miRNA assay were purchased from System Biosciences (Mountain View, CA).

### Statistical Analysis

The results are presented as the mean values ± SE. Data of patient specimens are presented graphically with median and interquartile range noted. Data were analyzed using analysis of variance or a Student’s *t* test with correction for data sets with unequal variances. Age-adjusted t-test is taken from a linear model including age as a covariate. Data were analyzed on a log 2 scale as appropriate.

### Conclusions

In summary, we found that miR-145 is downregulated in glial tumors by a methylation-independent mechanism. In addition, we demonstrated that the inhibitory effect of miR-145 on glioma cell migration and invasion and GSC migration was at least partly mediated by decreasing the expression of the novel miR-145 target, CTGF which further decreased the expression of SPARC. These results suggest that miR-145 and CTGF can act as potential novel anti-migration therapeutic targets in GBM.
